# Tuberculosis recurrence in smear-positive patients cured under DOTS in southern Ethiopia: retrospective cohort study

**DOI:** 10.1186/1471-2458-9-348

**Published:** 2009-09-18

**Authors:** Daniel G Datiko, Bernt Lindtjørn

**Affiliations:** 1 Centre for International Health, University of Bergen, Overlege Danielsens Hus, Årstadveien 21, 5009 Bergen, Norway; 2 Southern Nations, Nationalities, and Peoples' Regional Health Bureau, PO Box 149, Hawassa, Ethiopia

## Abstract

**Background:**

Decentralization of DOTS has increased the number of cured smear-positive tuberculosis (TB) patients. However, the rate of recurrence has increased mainly due to HIV infection. Recurrence rate could be taken as an important measure of long-term success of TB treatment. We aimed to find out the rate of recurrence in smear-positive patients cured under DOTS in southern Ethiopia.

**Methods:**

We did a retrospective cohort study on cured smear-positive TB patients who were treated from 1998 to 2006. Recurrence of smear-positive TB was used as an outcome measure. Person-years of observation (PYO) were calculated per 100 PYO from the date of cure to date of interview. Kaplan-Meier and Cox-regression methods were used to determine the survival and the hazard ratio (HR).

**Results:**

368 cured smear-positive TB patients which were followed for 1463 person-years. Of these, 187 patients (50.8%) were men, 277 patients (75.5%) were married, 157 (44.2%) were illiterate, and 152 patients (41.3%) were farmers. 15 of 368 smear-positive patients had recurrence. The rate of recurrence was 1 per 100 PYO (0.01 per annum). Recurrence was not associated with age, sex, occupation, marital status and level of education.

**Conclusion:**

High recurrence rate occurred among smear-positive patients cured under DOTS. Further studies are required to identify factors contributing to high recurrence rates to improve disease free survival of TB patients after treatment.

## Background

The World Health Organization (WHO) recommends directly observed treatment short-course (DOTS) to control tuberculosis (TB). It advocates early case detection and prompt treatment to ensure long-term success by reducing transmission, recurrence (relapse or reinfection) and death [1].

Decentralized DOTS implementation has increased the number of successfully treated TB patients [2-5]. However, in some countries, the incidence of TB has increased, as has the risk of defaulting, failure, death and recurrence, mainly because of the HIV epidemic [6,7]. Therefore, recurrence and death in successfully treated TB patients could be taken as an important measure of the efficacy of TB treatment. However, there are no routines in monitoring TB patients after completing treatment.

Post-treatment studies reported high recurrence rate in TB patients (36%) after 22 months of follow-up [8]. The recurrence rates were high among patients infected with HIV infected and multidrug resistant (MDR) TB (cases with strains resistant at least to isoniazid and rifampicin) [8-10]. This may increase TB incidence and reduce the treatment success [11,12].

In Ethiopia, the success of TB control is affected by the shortage of health workers to conduct case finding and treatment supervision [4]. In such settings, poor treatment adherence and extended treatment regimen could compromise the long-term efficacy of TB treatment by increasing the rate of recurrence, transmission of infection and emergence of drug resistance [13].

To our knowledge, no follow-up study has been conducted in Ethiopia to determine recurrence rates in cured smear-positive TB patients. The aim of the study was to find out the rate of recurrence through community based follow-up of smear-positive TB patients cured under DOTS.

## Methods

### Study area and population

**Figure 1:**
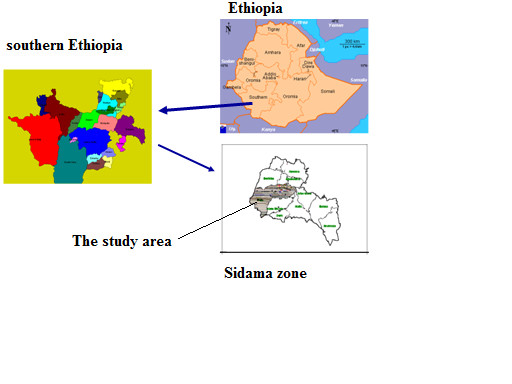
**Map of the study area in Sidama zone in the southern Ethiopia**.

We did this study in Dale and Wonsho districts of Sidama zone in the southern Ethiopia (Figure 1). It is a densely populated agrarian community (with a population of 296, 811). DOTS was started in 1996 [14] and six health facilities were providing TB service in the study area. Trained general health workers administer directly observed treatment. Standard recording and reporting formats were used in the health facilities and the districts. District TB programme experts regularly checked the completeness and accuracy of the recording in the unit TB register. The estimated prevalence of TB in the study area was 643 per 10^5 ^population; and the incidence of smear-positive cases was168 per 10^5 ^population for 2006 [4]. The case detection, cure and treatment success rates were 41%, 58% and 76% respectively. The sputum conversion rate at second month of follow-up was 83% (unpublished report from the study area).

### Study design

This was a retrospective cohort study based on TB patients that were registered in unit TB registers in the health facilities providing DOTS. We enrolled new and retreatment cases that were reported cured from 1998 to 2006 through house-to-house visit.

### Case definition, treatment duration and outcome

TB patients who had productive cough for two weeks or more with at least two positive sputum smears or one positive smear and x-ray findings consistent with active PTB were classified as smear-positive pulmonary TB cases.

TB patients received two months intensive phase and six months continuation phase treatment. Follow-up sputum examination was done at the end of 2^nd^, 5^th ^and 7^th ^months of treatment. A smear-positive TB patient who had negative sputum smear result in the last month of treatment and on at least one previous occasion (2^nd ^or 5^th ^month) was reported as *cured*. The term recurrence was used to indicate rediagnosis of smear-positive TB in patients who were reported cured [8].

### Data collection

From unit TB register in health institutions in the two districts, we obtained the list of smear-positive TB patients who were declared cured from 1998 to 2006. We collected information about unit register number, name, age, sex, address, TB category, smear result and the treatment outcome. The data was crosschecked with the district TB register that contained the list of the patients treated in the health institutions in the districts. Trained health extension workers conducted house-to-house visits, and collected data from the TB patients or their households. They collected the information if the TB patient were alive, had symptoms of TB and registered the date of the interview using structured questionnaire. The data collection was done from September 2007 to February 2008. HIV results were not available for TB patients enrolled in our study.

### Data analysis

We used SPSS 14 for Windows for data entry and analysis. We described the patients by age, sex, TB category, marital status, level of education and occupation. The outcome measure was recurrence of TB. Person-years of observation (PYO) were calculated from the date of cure to date of interview.

We used the Kaplan-Meier method to find out the event-free survival and the log-rank test for the statistical significance. Cox-regression method was used to determine the hazard ratio (HR) and 95% Confidence interval (95%CI). Recurrence rate was calculated as the number of recurrences per 100 PYO. P-value less than 0.05 was considered significant.

### Ethical clearance

The Ethical Review Committee of the Regional Health Bureau approved this study. After explaining the aim of the study, we obtained informed consent from the study participants or head of the household. Patients with recent history of cough and other symptoms suggestive of TB were advised to visit health posts for sputum collection by health extension workers or to visit diagnostic health institutions for examination.

## Results

Of the 397 smear-positive TB patients registered, 368 (92.7%) were followed. Incomplete information was obtained for 29 (7.3%) of which 8(2.0%) had moved to other districts. However, no difference was observed by age, sex, and TB category compared o the patients we enrolled.

Of the 368 smear-positive TB patients which were followed, 187 patients (50.8%) were men, 277 patients (75.5%) were married, 157 (44.2%) were illiterate, and 152 patients (41.3%) were farmers (Table 1). 368 cured smear-positive TB patients were followed for 1463 person-years. 15 of 368 smear-positive patients had recurrence. The mean (median) duration of follow-up was 3.87 (4.0) years. The rate of recurrence was 1 per 100 PYO (0.01 per annum). Recurrence was not associated with age, sex, occupation, marital status and level of education (Table 2).

**Table 1:** Socio-demographic characteristics of cured smear-positive patients in southern Ethiopia from 1998 - 2006

**Variables**	**number**	**Percent (%)**
**Sex**		
Male	187	50.8%
Female	181	49.2%
**Marital status**		
Single	90	24.5%
Married	268	73.0%
Divorced/widowed	9	2.5%
Missing	1	
**Level of education**		
Illiterate	157	44.2%
1 - 4	57	16.1%
5 - 8	120	33.8%
9 +	21	5.9%
Missing	13	
**Occupation**		
Student	64	17.4%
Farmer	152	41.3%
Housewife	35	9.5%
Merchant	16	4.3%
Others	101	27.4%
**Current status**		
New	364	98.9%
Retreatment	4	1.1%

**Table 2:** Factors predicting recurrence in cured smear-positive tuberculosis patients in southern Ethiopia from 1998 - 2006

**Variables**	**Recurrence**		PYO*	**Recurrence rate per 100PYO**	**Crude HR (95%CI)**^†^	**P - value**
	**Yes**	**No**				
**Age (in years)**						
< 15	0	28	122	0.0	1.0	
≥15	14	324	1330	1.1	0.0 (0.0 - 170)	0.5
**Sex**						
Female	5	176	710	7.0	1.0	
Male	10	177	753	1.3	1.8 (0.6 - 5.5)	0.3
**Level of education**						
Illiterate	5	152	629	0.8	1.0	
Literate	9	189	783	1.2	0.7 (0.2 - 1.9)	0.5
**Marital status**						
Never married	1	89	338	0.3	1.0	
Married	14	263	1118	1.3	03 (0.03 - 1.9)	0.2
**Occupation**						
Farmers	12	239	997	1.2	1.0	
Non farmers	3	114	466	0.6	1.9 (0.5 - 6.6)	0.3

* PYO - person-year observation

^† ^HR - hazard ratio, 95%CI - 95% confidence interval

## Discussion

The estimated recurrence rate in our study area was 1 per 100 PYO. This could be explained by HIV infection, MDR TB, reinfection due to high TB burden and inadequate treatment supervision and patient follow-up.

HIV infection increases the risk of infection, reinfection, recurrence and death. It also increased the workload by fuelling TB epidemic and affected the performance of TB programme [6]. In southern Ethiopia, the prevalence of HIV infection in the general population and TB patients was 3.8% and 17.5% respectively [15]. This could be one of the factors to explain the high recurrence rate in our setting. However, the role of HIV in recurrence requires further investigation.

Higher recurrence rates reported elsewhere, 8.6% in Vietnam after 19 months, 11% in India after two and half years and 36% in Kazakhstan after 22 months of follow-up [16-18] were attributed to MDR TB, poor treatment supervision and inadequate patient follow-up [8,9,11,12]. Though the prevalence of MDR TB in Ethiopia was believed to be low (1.6% in new and 12% in previously treated TB cases), 50% resistance to one or more drugs in re-treatment cases was reported) [19]. Similarly 7.7% resistance to at least one TB drug was reported from our study area [20]. This may also be one of the factors to explain the high recurrence rate in our setting.

Moreover, factors that affect the performance of TB programme (poor treatment supervision and failure to do follow-up sputum examination) and the patients' general condition could increase the recurrence rate. Inadequate treatment supervision, more pronounced during continuation phase when patients receive unsupervised treatment, reduces treatment adherence and increases the risk of treatment failure and MDR TB. This is worsened when the importance of treatment adherence is not well addressed during health education sessions [21]. Additionally, failure to conduct follow-up sputum examination reduces the chance of detecting failure cases (smear-positive at 5^th ^or 7^th ^month) without affecting the number of patients that complete treatment under DOTS. Thus, in routine practice where cure is based on smear microscopy, treatment failure can be missed.

The limitation of the study were using sputum microscopy for the diagnosis of recurrence in smear-positive patients that may have underestimated the rates of recurrence and lack of HIV test result to estimate the role of HIV in recurrence.

The significance of this study is more in settings with high TB and HIV prevalence. In such settings high disease transmission may maintain the burden of TB in the community. Yet, high recurrence rates in cured smear-positive TB patients should alert TB programme managers to identify the risk factors. The performance of TB programme could be improved by addressing factors that affect treatment adherence and increase the risk of MDR TB. TB patients could also benefit from the access to HIV prevention and control measures in high-risk patients to reduce recurrence and improve their long-term survival.

## Conclusion

The rate of recurrence in cured smear-positive TB patients was high in our setting. Further studies are required to identify risk factors for recurrence to improve the disease free survival of cured smear-positive TB patients.

## Competing interests

The authors declare that they have no competing interests.

## Authors' contributions

DGD supervised data collection. DGD and BL analyzed, interpreted the findings and prepared the drafts. All authors contributed to the final manuscript

## Pre-publication history

The pre-publication history for this paper can be accessed here:


